# Immune evasion of *Borrelia miyamotoi*: CbiA, a novel outer surface protein exhibiting complement binding and inactivating properties

**DOI:** 10.1038/s41598-017-00412-4

**Published:** 2017-03-22

**Authors:** Florian Röttgerding, Alex Wagemakers, Joris Koetsveld, Volker Fingerle, Michael Kirschfink, Joppe W. Hovius, Peter F. Zipfel, Reinhard Wallich, Peter Kraiczy

**Affiliations:** 1 Institute of Medical Microbiology and Infection Control, University Hospital of Frankfurt, Frankfurt, Germany; 2 Center for Experimental and Molecular Medicine, Academic Medical Center, Amsterdam, The Netherlands; 3 National Reference Center for Borrelia, Oberschleißheim, Germany; 4 Institute of Immunology, University of Heidelberg, Heidelberg, Germany; 5 Department of Infection Biology, Leibniz Institute for Natural Product Research and Infection Biology, Jena, Germany; 6 Friedrich Schiller University, Jena, Germany

**Keywords:** Immune evasion, Pathogens

## Abstract

*Borrelia (B.) miyamotoi*, an emerging tick-borne relapsing fever spirochete, resists complement-mediated killing. To decipher the molecular principles of immune evasion, we sought to identify determinants contributing to complement resistance. Employing bioinformatics, we identified a gene encoding for a putative Factor H-binding protein, termed CbiA (complement binding and inhibitory protein A). Functional analyses revealed that CbiA interacted with complement regulator Factor H (FH), C3, C3b, C4b, C5, and C9. Upon binding to CbiA, FH retained its cofactor activity for Factor I-mediated inactivation of C3b. The Factor H-binding site within CbiA was mapped to domain 20 whereby the C-terminus of CbiA was involved in FH binding. Additionally, CbiA directly inhibited the activation of the classical pathway and the assembly of the terminal complement complex. Of importance, CbiA displayed inhibitory activity when ectopically produced in serum-sensitive *B. garinii* G1, rendering this surrogate strain resistant to human serum. In addition, long-term *in vitro* cultivation lead to an incremental loss of the *cbiA* gene accompanied by an increase in serum susceptibility. In conclusion, our data revealed a dual strategy of *B. miyamotoi* to efficiently evade complement via CbiA, which possesses complement binding and inhibitory activities.

## Introduction

The genus *Borrelia* (*B.*) comprises a large number of host-associated spirochetes, all of which are transmitted by hematophagous arthropods and are the causative agents of relapsing fever as well as Lyme disease. Tick-borne relapsing fever (TBRF) spirochetes include *B. hermsii*, *B. parkeri*, *B. duttonii*, and *B. miyamotoi*. As an emerging human pathogen *B. miyamotoi* was first discovered in 1995 in Hokkaido (Japan)^1^ and is transmitted, in contrast to other TBRF spirochetes, by hard-bodied ticks, e.g. *Ixodes (I.) scapularis* and *I. pacificus* in North America, *I. ricinus* in Europe, and *I. persulcatus* in Asia. *B. miyamotoi* occurs sympatrically with *B. burgdorferi* s.l. in Asia^1^, North America^2^, and Europe^3^. Human cases were first reported in central Russia in patients with nonspecific febrile illness and symptoms similar to relapsing fever, including high-grade fever, headache, fatigue, chills, myalgia, arthralgia, and nausea^4^. Thereafter more clinical cases of *B. miyamotoi*-caused infections have been described in Europe^5^, Japan^6^ and North America^7–9^ leading to the implementation of a new entity termed hard tick-borne relapsing fever (HTBRF)^10^.

Until recently, *B. miyamotoi* was very difficult to culture under *in vitro* conditions, thus making it very difficult, if not almost impossible, to investigate innate immune evasion strategies of these enigmatic bacteria. By establishing an artificial medium supplemented with either human or bovine serum (Kelly-Pettenkofer medium) as a key prerequisite for *in vitro* studies, we were able to propagate *B. miyamotoi* for >40 passages without loss of viability, yielding cells densities of 7 × 10^6^/ml^11–13^.

Complement represents a central part of innate immunity and operates as a well-organized network comprising inactive precursors, fluid-phase and membrane-bound regulators and inhibitors^14–16^. The complement cascade is sequentially activated by three distinct pathways, the classical (CP), alternative (AP), and the lectin pathway (LP), thereby generating cleavage products that display multiple effector functions. These three pathways converge at the level of C3 by generating highly reactive C3b molecules, which function as opsonins and mark intruding microorganisms for phagocytosis. Upon progression, C5 convertases of the AP or CP/LP cleave C5 into C5b and C5a. Once C5b affixes to the microbial surface, the terminal sequence is initiated and the bacteriolytic terminal complement complex (TCC, C5b-9) is assembled by subsequent binding of C6, C7, C8 and several molecules of C9. All activation steps of the complement cascade are tightly controlled by a number of fluid phase and surface-attached regulators to avoid rampant activation on host cells. The 150 kDa Factor H (FH) protein is a key regulator of the AP, acting as cofactor for factor I-mediated degradation of C3b, and thereby supporting the dissociation (decay-accelerating activity) of the C3 convertase of the AP^16, 17^. FH is organized into 20 individually folding complement control protein (CCP) domains of which the N-terminally located CCP domains 1–4 are responsible for decay-accelerating and cofactor activity^18, 19^.

Relapsing fever spirochetes have developed numerous sophisticated mechanisms to successfully overcome innate immunity, in particular complement, by binding distinct complement regulators of the AP, CP, and LP via various outer surface proteins known to contribute to serum resistance^20–27^. More recently, we showed that *B. miyamotoi* survives in the presence of human serum, implicating that this particular emerging borrelial species possess determinants to overcome complement-mediated killing^11–13^. In this study, we set out to elucidate the underlying principles of complement resistance of *B. miyamotoi* and to identify and characterize factor(s) responsible for the interaction with human complement.

## Results

### Identification of a potential complement-interacting protein of *B. miyamotoi* HT31

To identify potential complement interacting protein(s) of *B. miyamotoi* a nucleotide Basic Local Alignment Search Tool (BLAST) search was conducted, using DNA sequences from *B. miyamotoi* FR64b deposited in GenBank. Comparative sequence analysis identified a gene (Accession number KF031443.1) that exhibited 70% identity at the DNA level to the gene encoding the FH-binding BhCRASP-1 protein of *B. hermsii* HS1^25^ and 51% identity on the protein level (Supplementary Figure S1). This gene encodes a putative outer surface protein with an estimated molecular weight of 21 kDa and has recently been annotated (Accession number CP004237.1) as a putative FH-like protein A (FhbA) of *B. miyamotoi* strain FR64b^28^. Primers generated were used for the amplification of the respective gene, lacking the signal sequence as well as the lipidation motif, by PCR and the resulting DNA fragments were cloned into the expression vector pQE-30 Xa, following digestion with the appropriate restriction endonucleases. To investigate differences in the complement-binding capabilities between the putative FH-binding protein of *B. miyamotoi* and additional homologous complement-interacting proteins from tick-borne and louse-borne relapsing fever spirochetes, e.g. *B. parkeri* (BpcA), *B. turicatae* (BtcA), and *B. recurrentis* (HcpA) a comparative analysis was performed^20, 26, 29, 30^ (Supplementary Figure S1). CspA from the Lyme disease spirochetes *B. burgdorferi* and BGA71 from *B. bavariensis* were included as additional controls^29, 31, 32^. All proteins were produced with a N-terminal His_6_-tag and purified via Ni-NTA affinity chromatography.

### Interaction of borrelial proteins with complement regulator FH and C4BP

**Figure 1:**
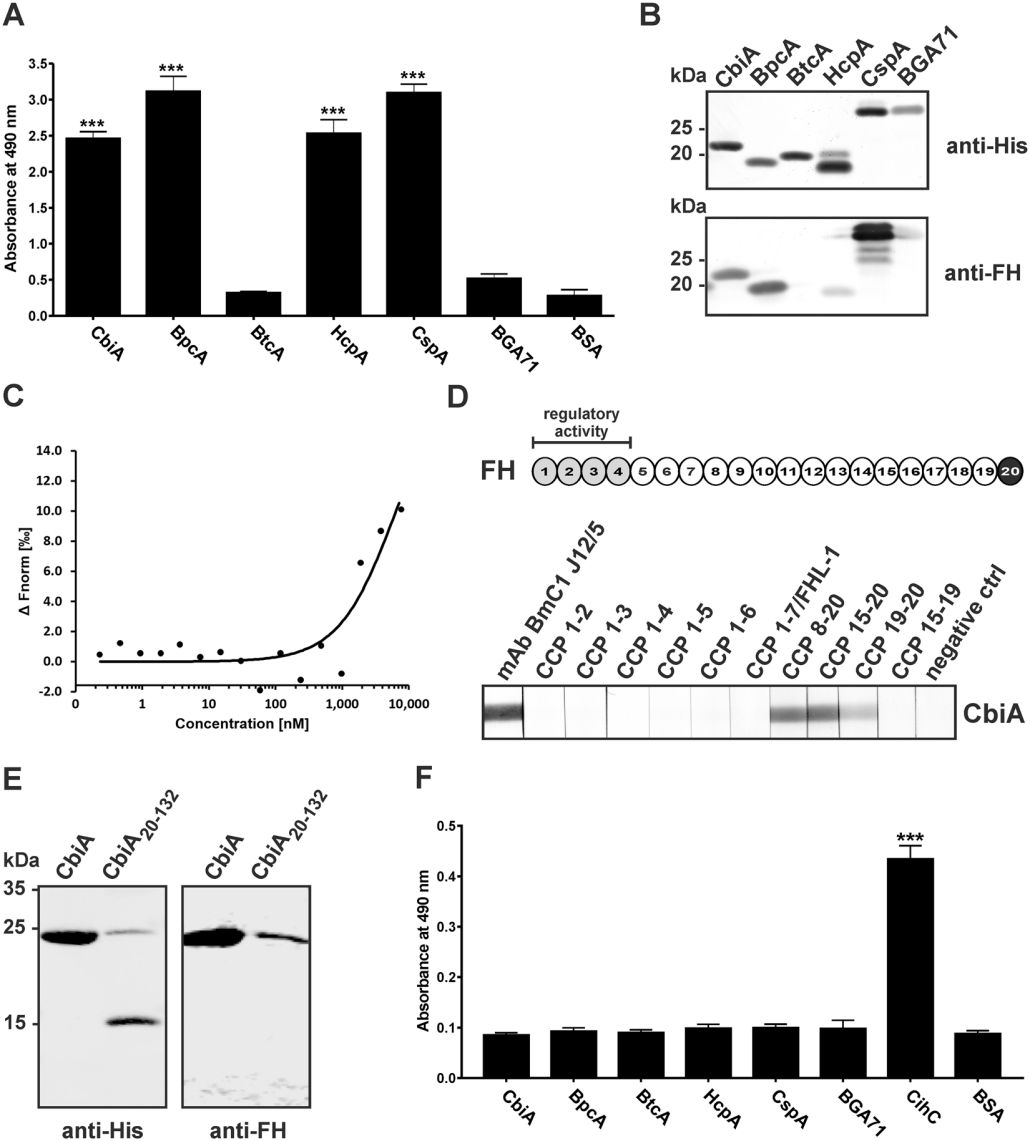
Binding of FH and C4BP to borrelial proteins and mapping of the interacting region in FH and CbiA. Binding of recombinant proteins to FH (**A**) and C4BP (**B**) was assessed by ELISA. Microtiter plates were coated with 500 ng His_6_-tagged proteins and incubated with FH or C4BP (5 µg/ml each). Bound FH and C4BP was detected using an anti-FH and anti-C4BP antiserum, respectively. All experiments were performed at least three times, with each individual test carried out in triplicate. **p ≤ 0.01, ***p ≤ 0.001, one-way ANOVA with Bonferroni post test. Far Western blot analysis of recombinant proteins (**B**). Proteins (500 ng each) were separated by SDS-PAGE and stained with silver or transferred to nitrocellulose. The membrane was incubated with NHS and subsequently probed with an anti-FH antiserum (lower panel). Binding of FH to CbiA (**C**). FH was labeled with NT-647 RED-NHS (NanoTemper technologies) and the interaction with CbiA was assessed in the fluid phase by microscale thermophoresis. The relative fluorescence in the thermophoresis phase has been plotted against the concentration of CbiA. The data shown are representative of three independent experiments. Localization of the binding domain in FH (**D**). Schematic representation of FH (upper panel). The CCP domains 1–4 are in light grey and the interacting domain is in black with white font. Mapping of the CbiA interacting domain in FH by Far Western blotting (lower panel). Purified recombinant CbiA was separated by SDS-PAGE, and transferred to nitrocellulose. The membrane strips were incubated with different constructs of FH (CCP1-2, CCP1-3, CCP1-4, CCP1-5, CCP1-6, CCP8-20, CCP15-20, CCP19-20, and CCP15-19), CCP1-7/FHL-1, mAb BmC1 J12/5, and with the secondary Ab (negative ctrl). Bound proteins were visualized using polyclonal anti-FH antibody. (**E**) Localization of the FH interacting domain in CbiA. His_6_-tagged CbiA and deletion mutant CbiA_20–132_ (500 ng each) were separated by SDS-PAGE and transferred to nitrocellulose. Membranes were probed with an anti-His_6_ antibody (upper panel) or incubated with NHS and subsequently probed with an anti-FH antiserum (lower panel). The full-length versions of (**B,D** and **E**) are presented in Supplementary Figure S4.

**Figure 2:**
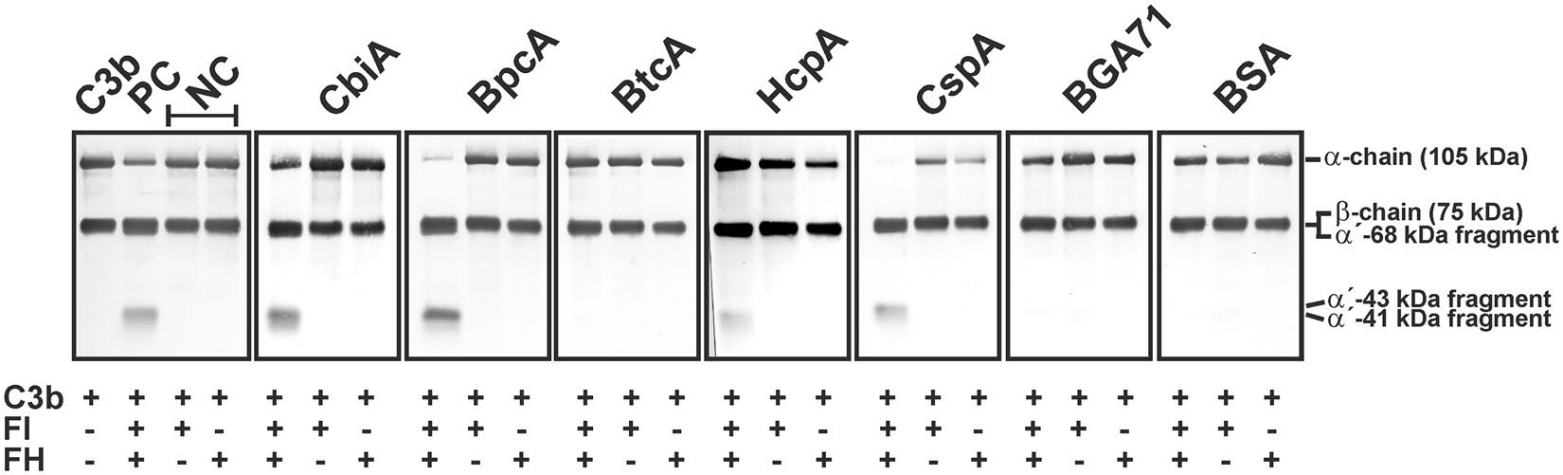
Analysis of functional activity of FH bound to borrelial proteins. Recombinant proteins immobilized to microtiter plates were used to capture FH. After extensive washing, C3b (100 ng/ml) and FI (200 ng/ml) were added and the mixture was incubated for 30 min at 37 °C. For control purposes, all reactions were also performed in the absence of FH and FI. Subsequently, samples were boiled for 5 min, subjected to 12.5% SDS-PAGE and transferred onto a nitrocellulose membrane. The various C3b degradation products were visualized by Western blotting using a polyclonal goat anti-human C3 antiserum. As additional controls, reaction mixtures containing purified C3b, C3b and FI were incubated with (+) or without (−) purified FH, respectively. The mobility of the α’- and the β-chain of C3 and the cleavage products of the α’-chain the α’68,’ α’46 and α’43 fragments are indicated. NC, negative controls; PC, positive control. A full-length version of the figures is presented in Supplementary Figure S5.

Recruitment of FH is a prerequisite for relapsing fever and Lyme disease spirochetes to evade complement-mediated killing^20, 25, 26, 32–36^. Assuming that the FhbA-like protein of *B. miyamotoi* HT31 also displays similar complement binding activities, we first investigated binding of FH to the *Borrelia*-derived proteins. The FhbA-like protein, designated as CbiA (complement binding and inhibitory protein A) was shown to bind to FH (Fig. 1A). As expected, binding of FH could also be detected with BpcA, HcpA, and CspA, while BtcA and BGA71 served as negative controls and did not bind FH^26, 29^. In addition, Far Western blotting was conducted as an independent approach to confirm interaction of the recombinant proteins with FH. As depicted in Fig. 1B, binding of CbiA, BpcA, and CspA to FH could clearly be demonstrated, while HcpA only weakly bound FH. Next, we sought to quantify the interaction of CbiA with FH by employing microscale thermophoresis. As shown in Fig. 2C, CbiA displayed a binding affinity of 6589.38 nM ± 1.52 to FH.

To gain further insight into the CbiA-FH interaction, we aimed to map the CbiA binding region within FH, using a number of truncated FH constructs as prey molecules for Far Western blotting. CbiA strongly bound constructs representing C-terminal domains CCP 8-20 and 15-20 and, although weakly, also bound to a construct comprising CCP domains 19 and 20 (CCP19-20) (Fig. 1D). In contrast, no binding to CbiA was detected when using constructs representing N-terminal CCPs of FH, e.g. CCP1-2, CCP1-3, CCP1-4, CCP1-5, and CCP1-6, CCP1-7/FHL-1 as well as C-terminal CCP15-19, suggesting that domain 20 is responsible for binding of FH to CbiA.

Previously, the involvement of higher ordered structural elements, in particular coiled coil domains in the formation of the FH interacting site has been reported from the *B. hermsii* FhbA protein^37^. To demarcate the FH binding site, we first sought to calculate the probability of CbiA to form coiled coils by using COILS^38^ (Supplementary Figure S2). Secondary structure prediction for CbiA revealed at least three putative α-helical regions of which a proposed coiled coil domain, located at the C-terminus, was deleted because the C-termini of both FhbA and BhCRASP-1 of *B. hermsii* are proposed to play a role in binding of FH^25, 37^. Purified CbiA and the truncated CbiA_20–132_ lacking 62 aa at the C-terminus were used for Far Western blot analysis with NHS as a source of FH. As shown in Fig. 1E, deletion of 62 aa at the C-terminus completely abrogated binding of FH to CbiA, suggesting that this particular region within CbiA is responsible for the interaction with FH. Regarding expression of the truncated CbiA_20–132_ (Fig. 1E, right lanes) low synthesis of a full-length form of CbiA (21 kDa) could be observed in addition to the truncated CbiA fragment (15 kDa). Translation readthrough of a “leaky” stop codon introduced at aa133 provides a plausible explanation for this finding.

When investigating interaction of borrelial proteins with complement regulator C4BP, we found that none of the proteins analyzed, including CbiA, bound to C4b except CihC of *B. recurrentis* which was used as positive control^27^ (Fig. 1F).

### FH retains its co-factor activity for FI-mediated C3b inactivation when bound to CbiA

Next, functional activity of FH bound to CbiA, BpcA, BtcA, HcpA, CspA, and BGA71 was assayed by FI-mediated cleavage of C3b. Recombinant proteins were coated onto microtiter plates, washed, then purified FI and C3b were added. The reaction mixtures were subjected to SDS-PAGE and the C3b cleavage products were detected by Western blotting. FH bound to CbiA, BpcA, HcpA, and CspA retained cofactor activity as indicated by the presence of C3b degradation products (68, 43, 41 kDa α’-chain) (Fig. 2). In the absence of FI, no iC3b fragments were detected when FH bound to the recombinant proteins. In addition, BtcA, BGA71, and BSA used as controls had no effect on cleavage of C3b. These experiments indicate that CbiA as well as BpcA, HcpA, and CspA exploit cofactor activity of FH to promote FI-mediated C3b degradation.

### Interaction of borrelial proteins with complement components

**Figure 3:**
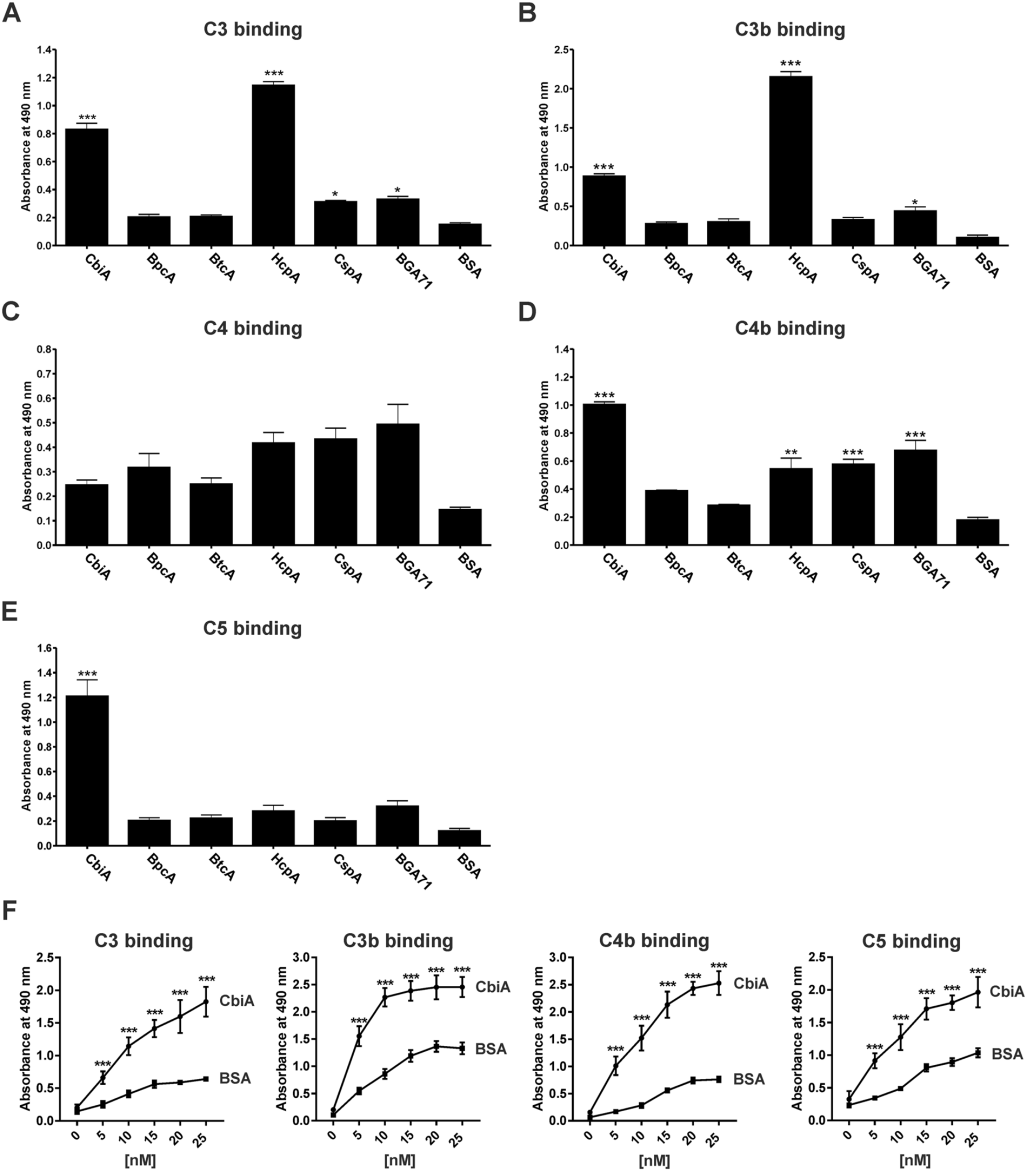
Binding of complement components to borrelial proteins. Binding of recombinant proteins to complement C3 (**A**), C3b (**B**), C4 (**C**), C4b (**D**), and C5 (**E**) was assessed by ELISA. Microtiter plates were coated with 500 ng His_6_-tagged proteins and incubated with the respective complement proteins (5 µg/ml each). Protein-protein complexes were detected using specific antibodies. Dose-dependent binding of C3, C3b, C4b, and C5 to CbiA (**F**). Microtiter plates were coated with 100 ng His_6_-tagged CbiA or 100 ng BSA and incubated with increasing concentrations with complement proteins and binding was analyzed with specific antibodies. Absorbance of each test was measured at 490 nm. Data represent means and SEM from three separate experiments, each performed at least in triplicate. Raw data were analyzed by one-way ANOVA with post hoc Bonferroni correction. ***P < 0.001, **P < 0.01, *P < 0.05.

In order to further explore the interaction of borrelial proteins with complement components involved in activation of the AP, CP, and LP, binding to C3, C3b, C4, C4b, and C5 was assayed using ELISA. Recombinant proteins immobilized to microtiter plates were incubated with the different complement components and protein-protein complexes were detected with specific antibodies. As demonstrated in Fig. 3, borrelial proteins exhibited different binding patterns for the distinct complement components. CbiA bound to C3, C4b, and C5, and to a lesser extent to C3b. HcpA of *B. recurrentis* also showed prominent binding to C3 and C3b but did not bind C5. A significant binding to C4b could also be detected for HcpA, CspA, and BGA71. Compared to BSA, none of the borrelial proteins bound to C4. These findings suggest that CbiA and HcpA interact with complement components at the initial phase of complement activation in multiple ways.

Having demonstrated interaction of CbiA with different complement components, we next sought to assess binding of C3, C3b, C4b, and C5 in more detail. As demonstrated in Fig. 3F, all complement proteins investigated bound to CbiA in a dose-dependent fashion.

### CbiA inhibits activation of the classical and terminal pathway

**Figure 4:**
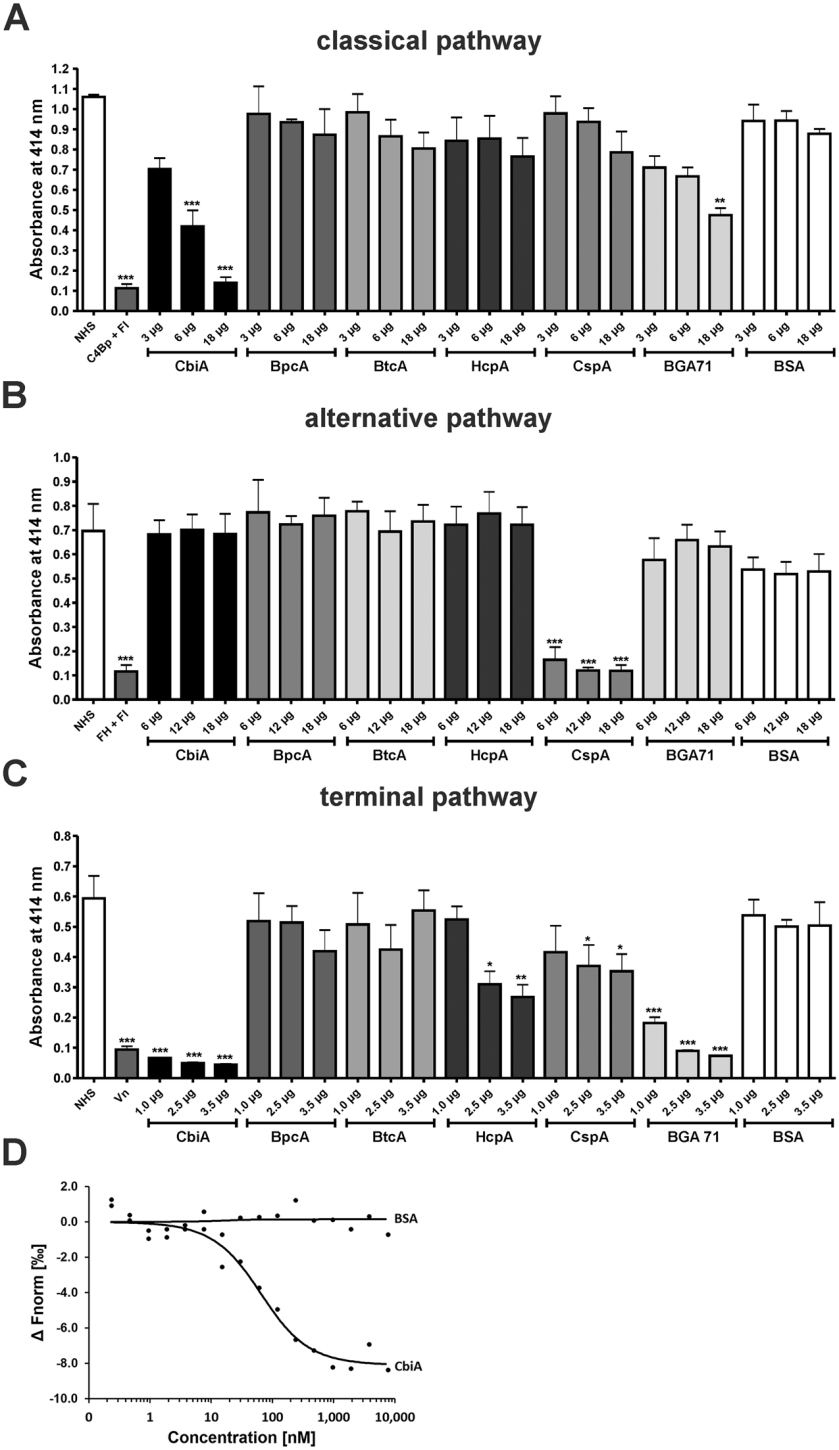
Borrelial proteins inhibit TCC deposition of the CP and TP. In order to analyze the inhibitory effect of borrelial proteins on the AP (**A**), CP (**B**), and TP (**C**) a hemolytic assay was employed. Amboceptor-sensitized sheep erythrocytes (CP) and rabbit erythrocytes (AP) were incubated with NHS or with NHS pre-incubated with purified proteins or BSA in either Mg-EGTA buffer (AP) or GVB^++^ buffer (CP). Inhibition of the TP was investigated using sheep erythrocytes pre-incubated with the C5b-6 complex. A reaction mixture containing C7, C8, and C9 was pre-incubated with increasing concentrations of purified proteins or BSA. Following incubation, erythrocyte lysis was detected at 414 nm. Means from three separate experiments are shown and error bars correspond to SD. Raw data were analyzed using one-way ANOVA (A and B). ***P < 0.001, **P < 0.01, *P < 0.05. Binding of C9 to CbiA (**D**). C9 was labeled with NT-647 RED-NHS (NanoTemper technologies) and the interaction with CbiA was assessed in the fluid phase. The relative fluorescence in the thermophoresis phase has been plotted against the concentration of CbiA. The data shown are representative of three independent experiments.

To corroborate that CbiA and the other *Borrelia*-derived proteins influence activation of the classical (CP), alternative (AP), and terminal pathway (TP), a cell-based hemolytic assay was employed. NHS was pre-incubated with increasing amounts (3, 6, and 10 µg) of the recombinant proteins and their complement inhibitory activity on erythrocytes was determined by determining the amount of hemolysis^29, 31, 32^. Regarding CP activation, CbiA significantly reduced hemolysis of amboceptor-sensitized sheep erythrocytes in a dose-dependent fashion, as did BGA71 at the highest concentration used (Fig. 4A). BpcA, BtcA, HcpA, CspA, and BSA did not inhibit CP activation. Investigating AP activation, none of the proteins significantly reduced hemolysis of rabbit erythrocytes with the exception of CspA (Fig. 4B). To confirm these results on AP activation, the formation of the C5b-9 complex (TCC) was determined using a commercial Wielisa®. Again, CspA strongly inhibited the AP, even at the lowest amount (6 µg) while all other proteins did not influence C5b-9 deposition under the same experimental conditions (Supplementary Figure S3).

Next, we wanted to gain further insight into the inhibitory capacity of CbiA on TCC formation. To this end, sheep erythrocytes were pre-incubated with the C5b-6 complex and increasing concentrations of recombinant proteins pre-incubated with C7, C8, and C9 were added. Following activation, CbiA, HcpA, CspA, and BGA71 protected erythrocytes from complement-mediated lysis in a dose-dependent fashion (Fig. 4C). Of note, among the borrelial proteins investigated, CbiA displayed the strongest inhibitory capacity^29, 32^. Collectively, these findings suggest that CbiA mainly affects CP- and TP-induced hemolysis.

Next, we sought to analyze the interaction of CbiA with complement components of the TP, C7, C8, and C9. Microscale thermophoresis experiments showed that CbiA only bound C9 with a K_d_ of 52.35 nM ± 0.63 (Fig. 4D) but not C7 or C8 (data not shown).

### CbiA is expressed in *B. miyamotoi* HT31

**Figure 5:**
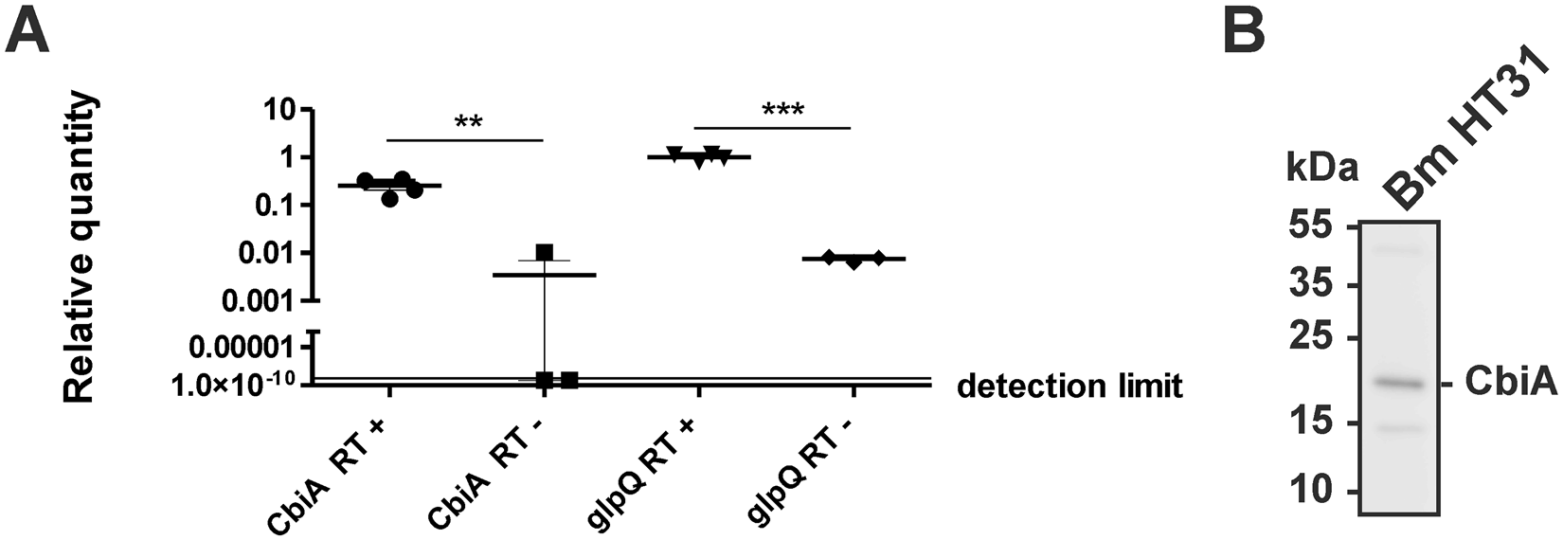
CbiA is expressed in *B. miyamotoi* HT31. (**A**) Expression of *cbiA* and *glpQ* was determined by RT-PCR. *B. miyamotoi* cells were grown at 33 °C and harvested at mid logarithmic growth phase. After transcription of isolated RNA the relative expression levels of the *cbiA* and the *glpQ* gene were determined. Each experiment was performed at least three times in triplicate and error bars represent ±SD. (**B**) Western blot analysis of *in vitro* grown spirochetes. 250 ng cell lysates were subjected to 4–20% SDS-PAGE and proteins were transferred to nitrocellulose. The membrane was probed with polyclonal rabbit anti-CbiA antibody (1:500) and protein complexes were visualized by a HRP-conjugated anti-rabbit antibody (1:10,000) using a Lumilight LAS4000. A full-length version is presented in Supplementary Figure S6.

Next, we analyzed *in vitro* expression of *cbiA* by using cultivated *B. miyamotoi* HT31 cells. RT-PCR analysis demonstrated that the CbiA encoding gene was expressed at the same level as the constitutively expressed *glpQ* gene in low-passaged *B. miyamotoi* at 33 °C (Fig. 5A). In addition, Western blot analysis also confirmed production of CbiA in cultivated *B. miyamotoi* HT31 (Fig. 5B), indicating that this particular protein was produced by *B. miyamotoi* when cultured in modified MKP-F medium.

### CbiA of *B. miyamotoi* is a surface-exposed protein

**Figure 6:**
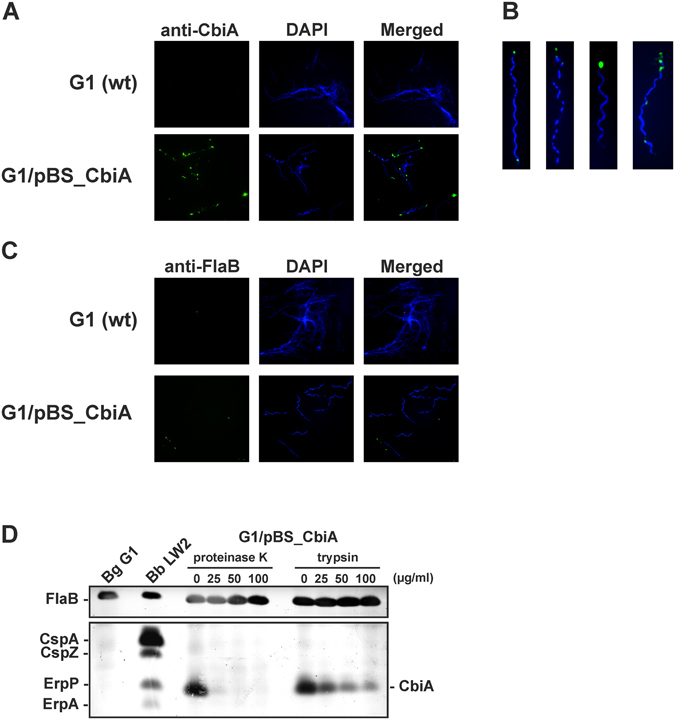
Surface exposure of CbiA in transformed *B. garinii* G1. (**A**) Surface localization of ectopically expressed CbiA was visualized by indirect immunofluorescence microscopy. Spirochetes (6 × 10^6^) were incubated with rabbit anti-CbiA antiserum (1:50) for 1 h at RT with gentle agitation. After fixation, glass slides were incubated with an appropriate AlexaFluor 488-conjugated secondary antibody. For visualization of the spirochetes in a given microscopic field, the DNA-binding dye DAPI was used. The spirochetes were observed at a magnification of 100 × objective. The data were recorded with an Axio Imager M2 fluorescence microscope (Zeiss) equipped with a Spot RT3 camera (Visitron Systems). Each panel shown is representative of at least 20 microscope fields. (**B**) *In situ* protease accessibility assay. Native spirochetes were incubated with or without proteinases, then lysed by sonication and total proteins were separated by SDS-PAGE. CbiA was identified by Far Western blot analysis using NHS as source of FH. Flagellin (FlaB) was detected with mAb L41 1C11. FH-binding proteins of *B. burgdorferi* LW2 (CspA, CspZ, ErpP, ErpA) are indicated on the left and the band corresponding to CbiA on the right. A full-length version is presented in Supplementary Figure S7.

To investigate the impact of CbiA on complement resistance of *B. miyamotoi -* and to overcome the technical limitations of genetic manipulation of this particular relapsing fever *Borrelia* species - we used the serum-sensitive *B. garinii* strain G1 for gain of function analysis of viable spirochetes producing CbiA. Following transformation, clones harboring the shuttle vector pBS_CbiA were assessed for the production and the surface-exposure of CbiA. To confirm localization of CbiA on the outer surface of transformed *B. garinii* cells, immunofluorescence microscopy was conducted using live spirochetes. As shown in Fig. 6A, transformed *B. garinii* G1/pBS_CbiA stained positive for CbiA. Strikingly, the majority of cells examined showed distribution of CbiA at the distal ends of the spirochetal cell (Fig. 6B). Integrity of the fragile borrelial outer membrane was confirmed by demonstrating lack of binding of antibodies directed against the periplasmic flagellar protein FlaB (Fig. 6C). In addition, surface localization of CbiA was examined by an *in situ* protease accessibility assay. After incubation of intact bacteria with trypsin and proteinase K, Far Western blotting was conducted to indirectly detect CbiA via binding of FH. CbiA was almost completely degraded after incubation of viable spirochetes with at least 25 μg/ml proteinase K (Fig. 6D). Upon treatment with trypsin, a more site-specific protease, degradation of CbiA could be detected at 25 µg/ml, while higher amounts of the same protease lead to a further reduction of the signal, supporting surface localization of CbiA on transformed spirochetes.

### CbiA facilitates complement resistance of *B. miyamotoi*

**Figure 7:**
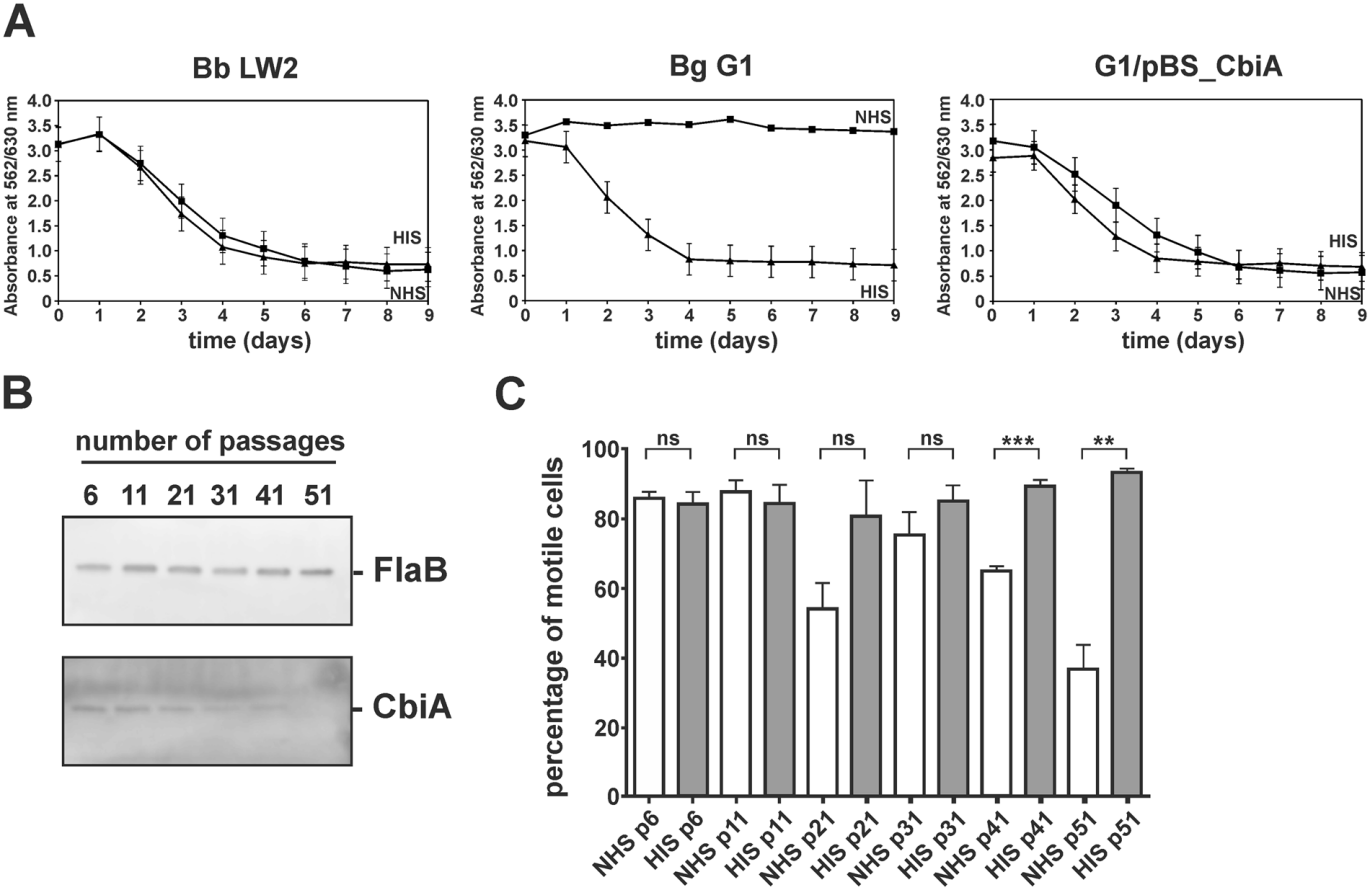
Serum susceptibility testing of spirochetes. (**A**) A growth inhibition assay was used to investigate susceptibility to human serum of *B. burgdorferi* (Bb) strain LW2, *B. garinii* (Bg) G1, and transformant G1/pBS_CbiA. Spirochetes were incubated in either 50% NHS (filled squares) or 50% HIS (filled triangles) over a cultivation period of 9 days at 33 °C, respectively. Color changes were monitored by measurement of the absorbance at 562/630 nm which is inversely proportional to the acidification of the culture medium. All experiments were performed at least three times, with each test conducted in triplicate with very similar results. For clarity, only data from one representative experiment is shown. Error bars represent ±SD. (**B**) Western blot analysis of spirochetes collected at different time points. Cell lysates (250 ng each) subjected to 4–20% SDS gels were analyzed by Western blotting to detect FlaB and CbiA using a monoclonal anti-FlaB antibody L41 1C11 (1:1000) and an anti-rabbit Ab (1:500), respectively. The full-length versions are presented in Supplementary Figure S8. (**C**) Serum susceptibility testing of spirochetes collected at different time points. Spirochetes were incubated in 50% NHS at 37 °C for 1 h. Dark-field microscopy were investigated for calculating the percentage of motile spirochetes. ns, not statistically significant. ***P < 0.001, **P < 0.01.

In order to investigate the impact of CbiA-mediated serum resistance, transformed spirochetes were challenged in 50% normal human serum (NHS) and heat-inactivated human serum (HIS) and viable spirochetes were monitored over an incubation period of 9 days by a serum susceptibility assay^39^. CbiA producing transformants as well as wild-type *B. burgdorferi* LW2 resisted complement-mediated bacteriolysis, as demonstrated by a continuous decrease in absorbance due to acidification of the medium. By contrast, growth of *B. garinii* G1 was completely inhibited in the presence of NHS. When using HIS instead of NHS, growth of all borrelial strains remained unaffected (Fig. 7A).

It has previously been shown that genetic rearrangements can occur during continuous *in vitro* cultivation of relapsing fever spirochetes, leading to the loss of the FhbA encoding gene of *B. hermsii* ^34^. Postulating that long-term propagation of *B. miyamotoi* may also results in genetic changes, we passaged spirochetes at least 51 times in MKP-F medium. At different time points (at passage 6, 11, 21, 31, 41, and 51), spirochetes were collected for detection of CbiA by Western blotting and for testing complement susceptibility by employing dark field microscopy. As depicted in Fig. 7B, CbiA disappeared over time and could not be detected after 51 passages. Spirochetes lacking CbiA displayed a serum-sensitive phenotype when challenged with NHS while no differences could be observed upon incubation of cells in HIS (Fig. 7C) indicating that CbiA confers resistance of *B. miyamotoi* to complement-mediated killing.

### Viable CbiA producing spirochetes recruit FH from human serum

**Figure 8:**
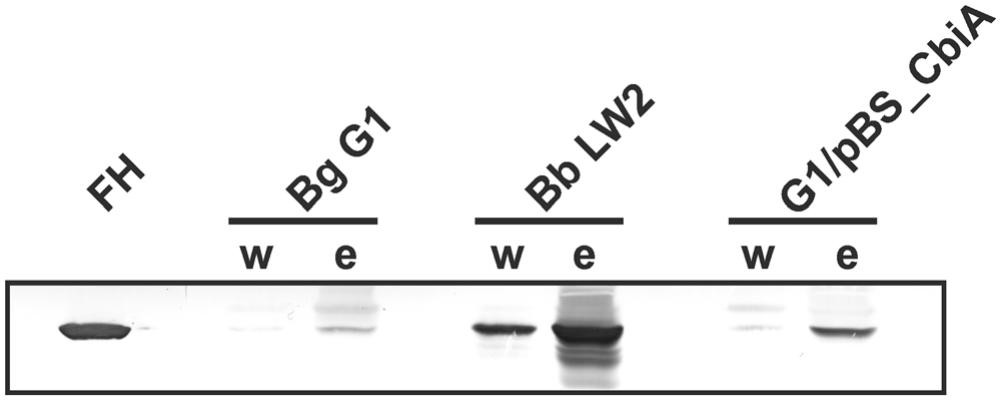
Binding of FH by *B. garinii* transformants. Serum adsorption assays were employed to detect binding of FH to viable spirochetes. Wild-type *B. garinii* (Bg) G1, *B. burgdorferi* (Bb) LW2, and transformant G1/pBS_CbiA (5 × 10^8^ cells each) were incubated in NHS-EDTA to prevent complement activation, washed, and then bound proteins were eluted using 0.1 M glycine (pH 2.0). Both the last wash (w) and the eluate (e) fractions obtained from each strain were separated by 12.5% SDS-PAGE and transferred to nitrocellulose. A polyclonal anti-FH antibody was used to detect FH by Western blotting. The full-length version is presented in Supplementary Figure S9.

Having demonstrated acquisition of FH by recombinant CbiA, a serum adsorption assay was utilized to confirm binding of this complement regulator to viable spirochetes. Consistent with the abovementioned data obtained with recombinant CbiA, serum-resistant G1/pBS_CbiA recruited FH from human serum, while serum-sensitive *B. garinii* G1 did not (Fig. 8). Of note, *B. burgdorferi* LW2, producing at least two FH-binding proteins, CspA and CspZ, bound higher amounts of FH compared to G1/pBS_CbiA indicating that the former strain is more potent in recruiting FH than the CbiA-producing strain.

## Discussion

Human pathogenic microorganisms exploit an arsenal of sophisticated mechanisms to efficiently evade one aspect of the innate immune response, overcoming the destructive effects of complement. For spirochetes, including *Borrelia* and *Leptospira*, recruitment of fluid phase complement regulators FH and C4BP plays a pivotal role in inactivating complement at the central point, namely the activation of C3^40, 41^. More recently, it has been shown that distinct outer surface proteins, e.g. BBK32, CspA, BGA66, and BGA71 of *Borrelia* interfere with certain complement components to inhibit CP activation by binding to C1r or to terminate assembly of the bacteriolytic complement complex by the interaction with C7, C8, and C9^29, 31, 32, 42^. Here, we identified for the first time an outer surface protein of the relapsing fever spirochete *B. miyamotoi* which exhibits multiple complement inhibitory activities as it binds to FH, C3, C3b, C4, C4b, C5, and C9 and thereby potentially blocks AP, CP, and TP activation. Due to its multifunctional properties, we propose to name this particular molecule “complement binding and inhibitory protein A” or CbiA.

Comparative sequence analysis revealed that CbiA exhibits considerable similarities to BhCRASP-1 of *B. hermsii* HS1 and to FhbA of *B. hermsii* YOR at the protein level^21, 25, 36^. Despite these similarities, all three molecules differ in their functional properties to recruit complement regulator FH. While CbiA and BhCRASP-1 exclusively bind FH via the C-terminal domain CCP20^25^, binding of FhbA was primarily mediated by CCP domains 1–7 of FH/FHL-1 as well as by CCP16-20 of FH^34^ (Fig. 1C). These data suggest that CbiA is functionally more closely related to BhCRASP-1 than to FhbA (see below). Furthermore, potential CbiA-like proteins sharing identities between 40 to 54% could be identified using BLAST in other relapsing fever *Borreliae*, e.g. *B. parkeri*, *B. turicatae*, *B. coriaceae*, *B. crocidurae*, *B. persica*, *B. duttonii*, and *B. recurrentis.* In addition, preliminary secondary structure predictions revealed that CbiA, BhCRASP-1, and FhbA exhibit considerable differences in the content of α-helices and coiled coil domains suggesting that these proteins vary in their three dimensional structures (Supplementary Figure S2). It is tempting to speculate that structural alterations may account for the ability to selectively bind to distinct complement regulators as clearly demonstrated for BhCRASP-1 and FhbA, which are known to bind to FH and FHR-1 or FH and FHL-1, respectively^25, 34^. Notably, the C-terminal CCP19-20 domains of FH are considered a “hot spot of binding” for a number of microbial proteins^43^, including CbiA (Fig. 1D) and various borrelial FH-binding proteins^20, 25, 26, 44, 45^. Moreover, we showed that deletion of the proposed C-terminal domain in CbiA completely abrogates binding of FH (Fig. 1D and Supplementary Figure S2). This finding is in line with previous studies of BhCRASP-1, FhbA, BpcA, and HcpA, demonstrating that C-terminal truncations dramatically altered the binding capability of these borrelial proteins^20, 25, 26, 34^. Introducing additional C-terminal deletions resulted in low amounts of recombinant protein production using different *E. coli* strains, or proteins became almost insoluble and accumulated in inclusion bodies, making it impossible to use these constructs for consecutive analyses.

Elucidating the biological significance of the FH interaction, we showed that FH associated with CbiA, BpcA, HcpA as well as CspA and retains its cofactor activity to promote FI-mediated cleavage of C3b as indicated by the respective degradation products (Fig. 3A). These finding suggest that acquisition of FH by CbiA may play a role in the overall strategy developed by *B. miyamotoi* to overcome complement-mediated killing, in particular since spirochetes ectopically producing CbiA at the surface acquired serum-derived FH (Fig. 8). Due to technical limitations to achieve large amounts of *B. miyamotoi* cells required for the serum adsorption assays (approx. 2 × 10^9^ cells) binding of FH could not be investigated.

Assuming that binding of complement regulators is a key mechanism in evading complement attack, we showed that CbiA and HcpA of relapsing fever borreliae directly bound to additional components of the AP and CP (Fig. 3). Proteins displaying multiple binding properties for different complement components have recently been identified in *B. recurrentis* and *B. duttonii* (ChiC)^27^, *B. hermsii* (FhbA)^37^, *B. burgdorferi* (CspA)^31^, *Leptospira interrogans* (Lsa23)^46^, *Yersinia enterocolitica* (YadA)^47^, and *Moraxella catarrhalis* (UspA)^48^, most of which interact with FH and C4BP, while binding of FH and C3 splice products was reported for YadA and UspA. Apparently, inactivation of the CP mediated by the recruitment of C4BP does not play a role, as none of the analyzed proteins, including CbiA, were able to bind to this particular complement regulator (Fig. 1B). In addition, binding of C4b by CbiA, HcpA, CspA, and BGA71 did not support subsequent binding of C4BP (data not shown), thus inactivation of the CP by CbiA most likely follows other principles as previously shown for *B. recurrentis* and *B. duttonii* ^27^, and *B. burgdorferi* ^42^. Of importance, CbiA specifically inhibited activation of the CP and TP, while AP activation was not influenced despite binding of FH (Fig. 4). To explain obvious discrepancies between different assays, it should be mentioned that the AP required the highest concentration of NHS (6%), while the CP only needs 0.1% NHS and the TP uses purified complement components. The higher concentration of serum used in the AP results in a much more efficient activation of complement that is always accompanied by a higher concentration of TCC compared to the purified complement components used to investigate inhibition of the TP. In all probability, the inhibitory capacity of CbiA in the former assay might be overwhelmed by the efficient complement activation through the AP and the resulting amplification loop. Moreover, it is tempting to speculate that binding of other, as yet unidentified serum-derived proteins may counteract binding of CbiA, BpcA and HcpA to FH as shown for the FH/FHR-1 interaction of ErpA, ErpC, and ErpP, in which FH was displaced under physiological conditions by FHR-1^49–51^. More recently, a similar scenario has been reported for BGA71 of *B. bavariensis* ^29^ and the complement inhibitors Efb-C and Ecb of *Staphylococcus aureus* ^52^ that failed to inhibit complement upon activation of the AP, but efficiently inhibited activation of the terminal sequence (Fig. 4). Remarkably, CbiA displayed the strongest inactivation capacity on the CP compared to all other molecules investigated, perhaps owing to its binding preference to C4b and C5, although an additional interaction with C2 or components of the C1 complex as previously shown for the borrelial BBK32 protein^42^ cannot be excluded. However, the molecular mechanism behind CP inactivation remains to be determined.

The inhibitory capacity of CbiA on the assembly of the membrane attack complex, C5b-9, is of particular interest as this assay relies on purified complement components, suggesting that no additional serum-derived proteins have an impact on the interaction of CbiA with components of the TP. Moreover, binding to both, C5 and C9 may foster termination of the terminal sequence more efficiently compared to BGA66 and BGA71 interacting only with late complement components namely C7, C8, and C9^29^. Interestingly, HcpA of *B. recurrentis* has also been shown to significantly inhibit TP activation, but less efficiently so than CbiA. As HcpA does not bind C5, it is likely that the mode of interaction differs from that of CbiA and resembles the mechanism disclosed for CspA, BGA66, and BGA71.

Taken together, our findings reveal that CbiA acts as a powerful complement inhibitory molecule at different steps of the cascade by specifically targeting CP and TP activation. Indeed, the importance of CbiA for mediating serum resistance was clearly demonstrated by transformation of an initially serum-sensitive *B. garinii* strain (Fig. 7A). To underscore the data obtained with transformed spirochetes, we also demonstrated that wild-type *B. miyamotoi* cells expressed the CbiA-encoding gene *in vitro* in a manner comparable to the constitutively expressed *glpQ* gene (Fig. 5A). In Lyme disease spirochetes, as well as other relapsing fever *Borrelia*, extended *in vitro* cultivation leads to loss of plasmids and genetic rearrangements^34, 53, 54^ but nothing is known concerning plasmid maintenance of *B. miyamotoi*. Here, we showed for the first time that continuous passaging causes the loss of the *cbiA* gene (Fig. 7B) Whether this loss is due to loss of plasmids or changes in expression remains to be investigated. However, we showed that loss of *cbiA* in turn renders spirochetes highly susceptible to complement (Fig. 7C), further underlining the importance of this novel complement inhibitory factor of *B. miyamotoi*.

In conclusion, our findings revealed a novel strategy of *B. miyamotoi* to efficiently evade complement-mediated killing by producing an outer surface protein, CbiA which possesses multifactorial binding and inhibitory activities to human complement. The presented data strongly suggest that this molecule represents an important immune evasion molecule, which, by acting on different activation levels, efficiently protects *B. miyamotoi* from the antibacterial effects of complement-mediated killing.

## Materials and Methods

### Bacterial strains and culture conditions

*B. miyamotoi* HT31 was cultivated at 33 °C in a modified Kelly-Pettenkofer medium supplemented with 10% fetal calf serum (MKP-F) as described previously^12^. *B. burgdorferi* LW2 (skin isolate, Germany), *B. garinii* G1 (CSF isolate, Germany) as well as transformant G1/pBS_CbiA were grown at 33 °C in Barbour-Stoenner-Kelly (BSK) medium (Bio & Sell, Feucht, Germany) or BSK supplemented with 125 µg/ml kanamycin to mid-logarithmic phase (1 × 10^8^ cells/ml)^55^. *Escherichia coli* JM109 was grown at 37 °C in yeast tryptone broth supplemented with the appropriate antibiotics.

### Human sera, antibodies, and other reagents

Non-immune normal human serum (NHS) was obtained from healthy blood donors with no history of a borrelial infection and was also used as a source of FH. The respective consent documents and the procedure of blood collection were approved by the ethics committee at the University Hospital Frankfurt, Goethe-University Frankfurt (control number 160/10) and were performed strictly in accordance with the international guidelines and regulations (Declaration of Helsinki, 1964). All healthy blood volunteers provided written informed consent.

Complement proteins were either purchased from Complement Technology (Tyler, TX) or their generation has been previously described^51, 56, 57^. Polyclonal antibodies for the detection of complement components were obtained from Complement Technology, Merck (Darmstadt, Germany) or Quidel (San Diego, CA). Rabbit anti-SCR1-4 antiserum was used for detection of FHL-1^56^. MAb L41 1C11 was used for the detection of borrelial FlaB^58^ and a polyclonal anti-His antibody was from GE Healthcare, Freiburg, Germany. Unless otherwise stated, antibodies were used at the following final dilutions: 1:1,000 for L41 1C11, anti-SCR1-4, and anti-complement antibodies, 1:3,000 for anti-His.

### Generation and purification of recombinant proteins

Specific primers were used to generate vectors pQE CbiA (cbiA Bam and cbiA R0) and pQE BtcA (BtBam and BtSal) for production of His-tagged CbiA and His-tagged BtcA from *Borrelia turicatae* strain 91E135 (Supplementary Table S1). After digestion with restriction enzymes BamHI/SalI for BtcA and Bam/PstI for CbiA, the amplified DNA fragments were ligated into pQE-30Xa. For expression of His-tagged fusion proteins plasmids were transformed in *E. coli* JM109. The generation of His-tagged BpcA, HcpA, ChiC, CspA, and BGA71 from *B. parkeri* RML, *B. recurrentis* A17, *B. burgdorferi* LW2, and *B. bavariensis* PBi has been described previously^20, 26, 27, 29, 32^. Expression of recombinant proteins and purification by using Amintra Ni-NTA affinity resin (Expedeon, Cambridge, UK) for affinity chromatography have been described more recently^55^.

### Enzyme-linked immunosorbent assay

Microtiter plates were coated with His-tagged proteins (5 µg/ml each) over night at 4 °C. After blocking with Blocking buffer III BSA (AppliChem, Darmstadt, Germany) for 2 h at room temperature, 5 μg/ml of complement components C3, C3b, C4, C4b, C5 or complement regulators FH or C4BP in PBS was added and incubated for 1 h at room temperature. Bound proteins were detected with the appropriate primary antibody and protein complexes were identified using secondary horseradish peroxidase-coupled antisera. The reaction was visualized with 1,2-phenylenediamine dihydrochloride (Sigma-Aldrich, Taufkirchen, Germany) and absorbance was measured at 490 nm.

For competitive ELISA, microtiter plates were coated with His-tagged borrelial proteins as described above. After blocking, complement components FH and C4b as well as FH and C4BP, (5 µg/ml each) in 100 µl PBS were added and incubated for 1 h at room temperature. Bound protein complexes were detected using anti-C4 or anti-FH antibodies and antigen-antibody complexes were detected as described above.

For dose-dependent binding, microtiter plates were coated with 100 ng of CbiA and after blocking increasing concentrations (5, 10, 15, 20, and 25 nM) of complement components were added and antigen-antibody complexes were detected as described above.

### SDS-PAGE, Western blot, and Far-Western blot analysis

Whole cell lysates obtained from *B. burgdorferi* LW2, *B. garinii* G1, and transformant G1/pBS_CbiA (15 µg per lane), *B. miyamotoi* HT31 (2 µg per lane) or purified recombinant proteins (500 ng per lane) were subjected to 10% Tris/Tricine-SDS-PAGE or 4–20% SDS gels (Bio-Rad) under reducing conditions and transferred to nitrocellulose as previously described^59^. Membranes were digitalized by using a GS-710 densitometer and scanned images were processed by using Quantity One, version 4.2.1 (Bio-Rad).

### Cell-based hemolytic assays

To examine the inhibitory potential of borrelial proteins on the AP, CP, and the terminal pathway (TP) hemolytic assays were performed as described previously^29, 32^. In brief, 1 × 10^7^ amboceptor-sensitzed sheep erythrocytes (CP) or rabbit erythrocytes (AP) were incubated with either NHS or NHS pre-incubated with increasing concentrations of borrelial proteins or BSA. Sheep erythrocytes (1.5 × 10^7^ cells) pre-incubated with C5b-6 (1.5 µg ml^−1^) were used to assess inhibition of the TP. In parallel, C7 (2 µg ml^−1^), C8 (0.4 µg ml^−1^), and C9 (2 µg ml^−1^) were pre-incubated with or without increasing concentrations of borrelial proteins. Following addition of pre-incubated sheep erythrocytes with the pre-incubated borrelial proteins, sheep erythrocytes were sedimented and hemolysis was determined by measuring the absorbance of the supernatant at 414 nm.

### Complement activation assays

The inhibitory capacity of borrelial proteins on the alternative pathway was analyzed by Wielisa® (Euro Diagnostica, Malmö, Sweden). The alternative pathway were activated by lipopolysaccharides (LPS) (10 μg/ml) and deposition of the formed C5b-9 complex was assayed^60^. Reactions containing NHS were set to 100%. For inhibition, recombinant proteins as well as BSA (1–100 μg/ml each) were pre-incubated with 20% NHS for 15 min at 37 °C and thereafter added to the wells.

### Microscale thermophoresis

The binding of FH, C7, C8 and C9 to CbiA was evaluated using microscale thermophoresis as previously described^29, 31^. Briefly, FH (6.67 µM), C7 (10.8 µM), C8 (6.67 µM) and C9 (15.15 µM) were labeled with NT-647 RED-NHS (NanoTemper technologies). Dilutions of labeled complement components and non-labeled CbiA (1:2) ranging from 0.0002 to 76 µM were measured at 20% LED power and 80% MST power for 30 s in a Monolith NT.115 instrument (NanoTemper Technologies GmbH). All measurements were performed at RT and in triplicate.

### Functional assay for cofactor activity of FH

Cofactor activity of protein-bound FH was analyzed by measuring FI-mediated conversion of C3b to iC3b as described previously^51^.

### Construction of shuttle vector for ectopic production of CbiA in *B. garinii*

Shuttle vector pBSVA was used for ectopic production of CbiA in *B. garinii* G1. The respective CbiA encoding gene was amplified from *B. miyamotoi* HT31 by PCR using primers cbiA Bam and cbiA Sph (Supplementary Table 1). Amplicons generated were digested with the appropriate restriction endonucleases and cloned into pBSVA, yielding shuttle vector pBS_CbiA. The inserted sequences were subjected to nucleotide sequencing to ensure that no mutations have been introduced during PCR and subsequent cloning procedures. In addition, sequence alignments were performed to verify identity of the cloned sequences with their genomic counterparts using CLC Sequence Viewer 7.6.1 (Qiagen, Hilden, Germany).

### Generation and characterization of transformed *B. garinii* G1 strains

*B. garinii* strain G1 was grown in BSK medium and harvested at mid-logarithmic phase (1 × 10^8^ cells/ml). Preparation and transformation of electrocompetent cells as well as the selection of positive clones by microdilution have been described previously^55^. Ectopic expression of CbiA was verified by Western blotting and surface localization was confirmed by a *in situ* protease accessibility assays as well as immunofluorescence microscopy as described^50^. To avoid damage to the fragile borrelial outer membrane, intact bacteria were incubated with antibodies before being fixed onto glass slides.

### Real-time PCR analysis of the *cbiA* gene transcription in *B. miyamotoi*

Expression of *cbiA* in *B. miyamotoi* HT31 was assessed by qRT-PCR. Spirochetes from low-passage glycerol stocks grown for 7 days at 33 °C in MKP-F were harvested by centrifugation, washed three times with PBS and resuspended in the same buffer. RNA was isolated using the Nucleospin RNA II kit (Macherey-Nagel) according to manufacturer’s instruction. Isolated RNA was heated at 85 °C for 5 min, and a reverse transcriptase (RT) mix was added: 4 µl RT buffer (Promega), 2 µl dNTP mix (Invitrogen), 1 µl Oligo dT primer (Fermentas), 0.5 µl DTT (Invitrogen), 1.5 µl RNAse-free H_2_O, 0.5 µl M-MLV RT (200 U/ml, Promega) and 0.5 µl RNAse out (Invitrogen). As a negative control, RT was replaced by H_2_O. Reactions were then incubated at 23 °C for 10 min, at 42 °C for 60 min and finally at 95 °C for 3 min. qRT-PCR was performed by adding 1 µl of the reaction mixtures, 3.6 µl H_2_O, 5 µl Sensifast SYBR no-ROX (Bioline), and 0.4 µl 10 µM of the appropriate primers (glpQ FW and glpQ RV, cbiA FW and cbiA RV) (Supplementary Table 1). Samples were then incubated initially at 95 °C for 2 min followed by 50 cycles of 95 °C (5 s), 60 °C (10 s), 71 °C (10 s) on a Roche Lightcycler 480. Results were analysed using LinregPCR software^61^, and relative values were compared to the mean glpQ cDNA value. A ct of 50 was used as detection limit and was given to negative values. A melting curve was performed to check products, which were also investigated on 1% agarose.

### Binding of native FH to the borrelial surface

A serum adsorption assay was employed to assess binding of FH to viable spirochetes (5 × 10^8^ cells). After incubation with EDTA-treated NHS, surface-bound proteins were eluted and the last wash and the eluate fractions were separated by SDS-PAGE under non-reducing conditions. Following Western blotting, FH was detected with a polyclonal anti-FH antibody as described previously^35, 50^.

### Serum susceptibility testing of borrelial strains

Serum susceptibility of wild-type strains as well as transformed spirochetes to active human complement was assessed by a serum bactericidal assay^39, 62^. Briefly, spirochetes (1.25 × 10^7^) were incubated in BSK medium containing 50% NHS or 50% hiNHS for 9 days at 33 °C and the survival of bacterial cells was monitored by measuring the color shift of phenol red due to acidification of the medium. For data analysis the Gen5 software, Version 1.11.4 (BioTek Instruments) was used. Each experiment was conducted at least three times and means ± SD were calculated.

*Borrelia miyamotoi* HT31 was passaged weekly for one year, and passage 5, 10, 20, 30, 40 and 50 were stored in glycerol at −80 °C^12^. Next, glycerol stocks were cultured in MKP-F medium and diluted to 3 × 10^6^/ml. Twenty-five microliters of borrelial culture and 25 µl of NHS or HIS were added to four replicate wells, and incubated at 37 °C for 1 h. Next, 5 µl samples were investigated for percentage of motile spirochetes by dark-field microscopy as described previously^12^.

### Structural analysis

For calculation of predicted coiled coil domains and α-helices within CbiA, the COILS^38^ and ProtScale programs were used (www.expasys.org). COILS was performed with (2.5) and without weighting and windows of 14, 21, and 28 amino acid residues.

### Statistical analyses

Both the unpaired student's t-test and one-way ANOVA test with Bonferroni's multiple comparison post test were employed for statistical analysis using GraphPad Prism Version 4. Results were deemed statistically significant for the following p values: *P < 0.05, **P < 0.01, and ***P < 0.001.

## Electronic supplementary material

Supplementary Figures
