# Author Correction: Immune evasion of *Borrelia miyamotoi*: CbiA, a novel outer surface protein exhibiting complement binding and inactivating properties

**DOI:** 10.1038/s41598-019-42948-7

**Published:** 2019-05-02

**Authors:** Florian Röttgerding, Alex Wagemakers, Joris Koetsveld, Volker Fingerle, Michael Kirschfink, Joppe W. Hovius, Peter F. Zipfel, Reinhard Wallich, Peter Kraiczy

**Affiliations:** 1 Institute of Medical Microbiology and Infection Control, University Hospital of Frankfurt, Frankfurt, Germany; 2 Center for Experimental and Molecular Medicine, Academic Medical Center, Amsterdam, The Netherlands; 3 National Reference Center for Borrelia, Oberschleißheim, Germany; 4 Institute of Immunology, University of Heidelberg, Heidelberg, Germany; 5 Department of Infection Biology, Leibniz Institute for Natural Product Research and Infection Biology, Jena, Germany; 6 Friedrich Schiller University, Jena, Germany

Correction to: *Scientific Reports* 10.1038/s41598-017-00412-4, published online 22 March 2017

This Article contains errors.

The legend of Figure 1 is incorrect,

“Binding of FH and C4BP to borrelial proteins and mapping of the interacting region in FH and CbiA. Binding of recombinant proteins to FH (**A**) and C4BP (**B**) was assessed by ELISA. Microtiter plates were coated with 500 ng His_6_-tagged proteins and incubated with FH or C4BP (5 µg/ml each). Bound FH and C4BP was detected using an anti-FH and anti-C4BP antiserum, respectively. All experiments were performed at least three times, with each individual test carried out in triplicate. **p ≤ 0.01, ***p ≤ 0.001, one-way ANOVA with Bonferroni post test. Far Western blot analysis of recombinant proteins (**B**). Proteins (500 ng each) were separated by SDS-PAGE and stained with silver or transferred to nitrocellulose. The membrane was incubated with NHS and subsequently probed with an anti-FH antiserum (lower panel). Binding of FH to CbiA (**C**). FH was labeled with NT-647 RED-NHS (NanoTemper technologies) and the interaction with CbiA was assessed in the fluid phase by microscale thermophoresis. The relative fluorescence in the thermophoresis phase has been plotted against the concentration of CbiA. The data shown are representative of three independent experiments. Localization of the binding domain in FH (**D**). Schematic representation of FH (upper panel). The CCP domains 1–4 are in light grey and the interacting domain is in black with white font. Mapping of the CbiA interacting domain in FH by Far Western blotting (lower panel). Purified recombinant CbiA was separated by SDS-PAGE, and transferred to nitrocellulose. The membrane strips were incubated with different constructs of FH (CCP1-2, CCP1-3, CCP1-4, CCP1-5, CCP1-6, CCP8-20, CCP15-20, CCP19-20, and CCP15-19), CCP1-7/FHL-1, mAb BmC1 J12/5, and with the secondary Ab (negative ctrl). Bound proteins were visualized using polyclonal anti-FH antibody. (**E**) Localization of the FH interacting domain in CbiA. His_6_-tagged CbiA and deletion mutant CbiA_20–132_ (500 ng each) were separated by SDS-PAGE and transferred to nitrocellulose. Membranes were probed with an anti-His_6_ antibody (upper panel) or incubated with NHS and subsequently probed with an anti-FH antiserum (lower panel). The full-length versions of (**B,D** and **E**) are presented in Supplementary Figure S4.”

should read:

“Binding of FH and C4BP to borrelial proteins and mapping of the interacting region in FH and CbiA. Binding of recombinant proteins to FH was assessed by ELISA (**A**). Microtiter plates were coated with 500 ng His_6_-tagged proteins and incubated with FH (5 µg/ml). Bound FH was detected using an anti-FH antiserum. All experiments were performed at least three times, with each individual test carried out in triplicate. **) p ≤ 0.01, ***) p ≤ 0.001, one-way ANOVA with Bonferroni post test. Far Western blot analysis of recombinant proteins (**B**). Proteins (500 ng each) were separated by SDS-PAGE and transferred to nitrocellulose. The membrane was incubated with an anti-His_6_ antibody (upper panel) or NHS and subsequently probed with an anti-FH antiserum (lower panel). Binding of FH to CbiA (**C**). FH was labeled with NT-647 RED-NHS (NanoTemper technologies) and the interaction with CbiA was assessed in the fluid phase by microscale thermophoresis. The relative fluorescence in the thermophoresis phase has been plotted against the concentration of CbiA. The data shown are representative of three independent experiments. Localization of the binding domain in FH (**D**). Schematic representation of FH (upper panel). The CCP domains 1–4 are in light grey and the interacting domain is in black with white font. Mapping of the CbiA interacting domain in FH by Far Western blotting (lower panel). Purified recombinant CbiA was separated by SDS-PAGE, and transferred to nitrocellulose. The membrane strips were incubated with different constructs of FH (CCP1-2, CCP1-3, CCP1-4, CCP1-5, CCP1-6, CCP8-20, CCP15-20, CCP19-20, and CCP15-19), CCP1-7/FHL-1, mAb BmC1 J12/5, and with the secondary Ab (negative ctrl). Bound proteins were visualized using polyclonal anti-FH antibody. Localization of the FH interacting domain in CbiA (**E**). His_6_-tagged CbiA and deletion mutant CbiA_20-132_ (500 ng each) were separated by SDS-PAGE and transferred to nitrocellulose. Membranes were probed with an anti-His_6_ antibody (upper panel) or incubated with NHS and subsequently probed with an anti-FH antiserum (lower panel). Binding of recombinant proteins to C4BP (**F**). ELISA was conducted as described in (**A**) with C4BP detected using anti-C4BP antiserum. The full-length versions of figures 1**B**, **D**, and **E** are presented in Supplementary Figure S4.”

In addition, the legend of Figure 6 is incorrect,

“Surface exposure of CbiA in transformed *B. garinii* G1. (**A**) Surface localization of ectopically expressed CbiA was visualized by indirect immunofluorescence microscopy. Spirochetes (6 × 10^6^) were incubated with rabbit anti-CbiA antiserum (1:50) for 1 h at RT with gentle agitation. After fixation, glass slides were incubated with an appropriate AlexaFluor 488-conjugated secondary antibody. For visualization of the spirochetes in a given microscopic field, the DNA-binding dye DAPI was used. The spirochetes were observed at a magnification of 100 × objective. The data were recorded with an Axio Imager M2 fluorescence microscope (Zeiss) equipped with a Spot RT3 camera (Visitron Systems). Each panel shown is representative of at least 20 microscope fields. (**B**) *In situ* protease accessibility assay. Native spirochetes were incubated with or without proteinases, then lysed by sonication and total proteins were separated by SDS-PAGE. CbiA was identified by Far Western blot analysis using NHS as source of FH. Flagellin (FlaB) was detected with mAb L41 1C11. FH-binding proteins of *B. burgdorferi* LW2 (CspA, CspZ, ErpP, ErpA) are indicated on the left and the band corresponding to CbiA on the right. A full-length version is presented in Supplementary Figure S7.”

should read:

“Surface exposure of CbiA in transformed *B. garinii* G1. (**A**–**C**) Surface localization of ectopically expressed CbiA was visualized by indirect immunofluorescence microscopy. Spirochetes (6 × 10^6^) were incubated with rabbit anti-CbiA antiserum (1:50) (**A** and **B**) or with a mAb anti-FlaB antiserum (1:20) (**C**) for 1 h at RT with gentle agitation. After fixation, glass slides were incubated with an appropriate AlexaFluor 488-conjugated secondary antibody. For visualization of the spirochetes in a given microscopic field, the DNA-binding dye DAPI was used. The spirochetes were observed at a magnification of 100′ objective. The data were recorded with an Axio Imager M2 fluorescence microscope (Zeiss) equipped with a Spot RT3 camera (Visitron Systems). Each panel shown is representative of at least 20 microscope fields. Panel B shows enlarged images of individual cells producing CbiA at the poles (**D**) *In situ* protease accessibility assay. Native spirochetes were incubated with or without proteinases, then lysed by sonication and total proteins were separated by SDS-PAGE. CbiA was identified by Far Western blot analysis using NHS as source of FH. Flagellin (FlaB) was detected with mAb L41 1C11. FH-binding proteins of *B. burgdorferi* LW2 (CspA, CspZ, ErpP, ErpA) are indicated on the left and the band corresponding to CbiA on the right. A full-length version is presented in Supplementary Figure S7.”

