# Supplementary material for: Immune evasion of Borrelia miyamotoi: CbiA, a novel outer surface protein exhibiting complement binding and inactivating properties
Source: Sci Rep. 2017 Mar 22;7:303. doi: 10.1038/s41598-017-00412-4 (PMC5428533; doi:10.1038/s41598-017-00412-4)
Supplement: Supplementary file 1 — Supplementary Figures [file 41598_2017_412_MOESM1_ESM.pdf]

Supplementary Information

Immune evasion of *Borrelia miyamotoi*: CbiA, a novel outer surface protein exhibiting complement binding and inactivating properties

Florian Röttgerding[1], Alex Wagemakers[2†], Joris Koetsveld[2†], Volker Fingerle[3], Michael Kirschfink[4], Joppe W. Hovius[2], Peter F. Zipfel[5,6], Reinhard Wallich[4], and Peter Kraiczy[1*]

[1]Institute of Medical Microbiology and Infection Control, University Hospital of Frankfurt, Frankfurt, Germany
[2]Center for Experimental and Molecular Medicine, Academic Medical Center, Amsterdam, The Netherlands
[3]National Reference Center for Borrelia, Oberschleißheim, Germany
[4]Institute of Immunology, University of Heidelberg, Heidelberg, Germany
[5]Institute of Immunology, University of Heidelberg, Heidelberg, Germany
[6]Friedrich Schiller University, Jena, Germany

† A.W. and J.K. contributed equally to this study

Corresponding author: Dr. Peter Kraiczy, Institute of Medical Microbiology and Infection Control, University Hospital of Frankfurt, Paul-Ehrlich-Str. 40, D-60596 Frankfurt, Germany, Phone: +49-69-6301-7165, E-mail: Kraiczy@em.uni-frankfurt.de

Supplementary Figure S1

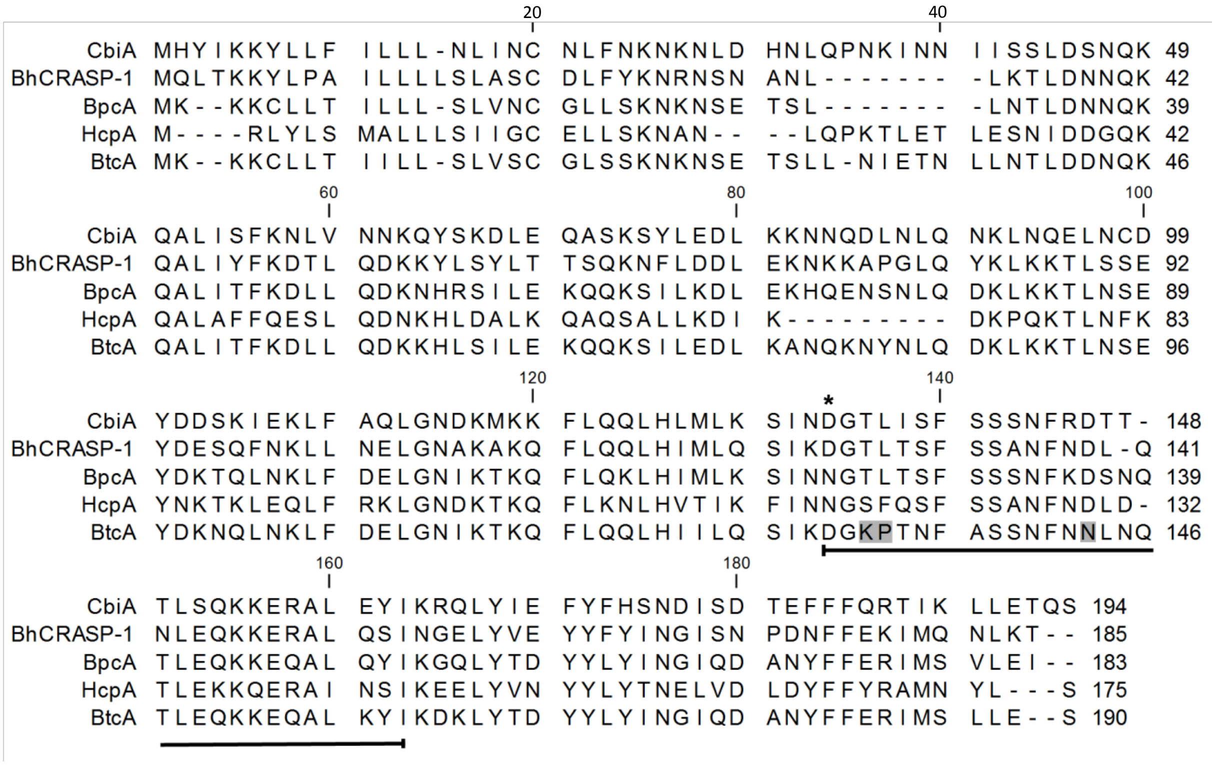

Alignment of proteins of relapsing fever spirochetes. Deduced amino acid sequences of CbiA, BhCRASP-1, BpcA, HcpA, and BtcA were aligned using CLC Sequence Viewer, Version 7.7 (Qiagen, Aarhus). Conserved residues are highlighted in light grey and the proposed FH-binding region derived from BhCRASP-1 is underlined. Asterisk indicates the introduced stop codon at position 133 to generate mutant $CbiA_{20\text{-}132}$ lacking 62 aa at the C-terminus.

Supplementary Figure S2

CbiA — alpha helix prediction / coiled coil prediction

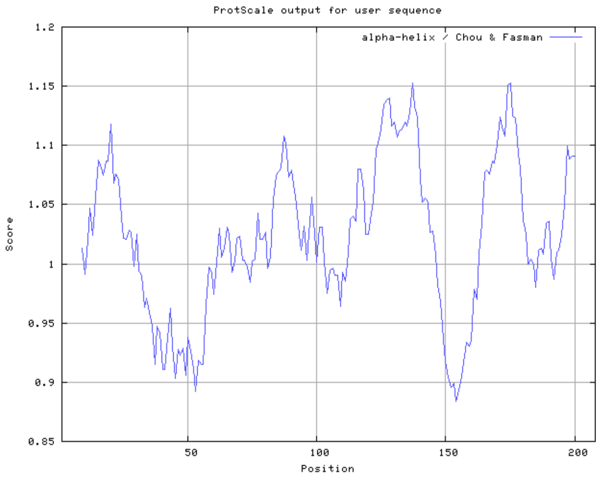

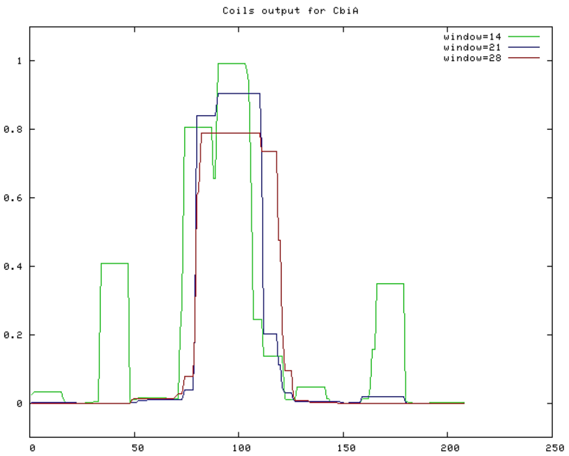

BhCRASP-1

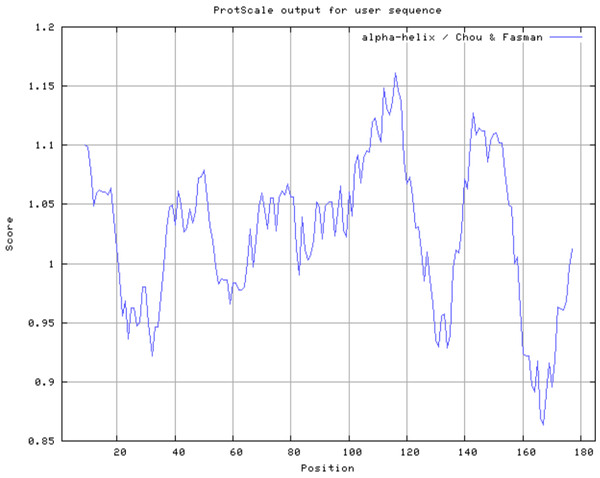

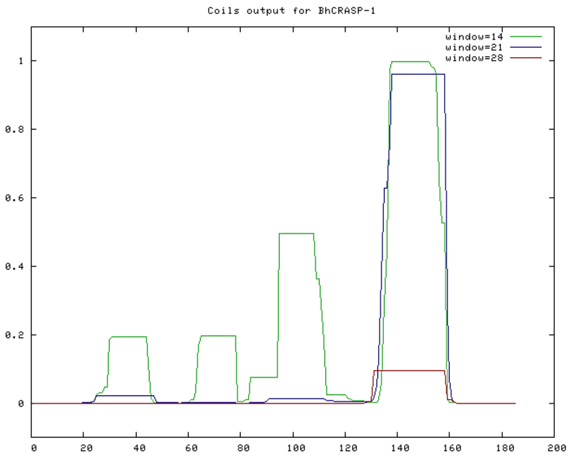

FhbA

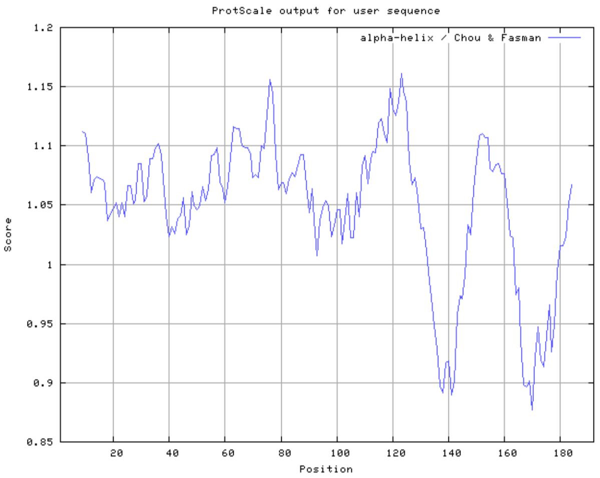

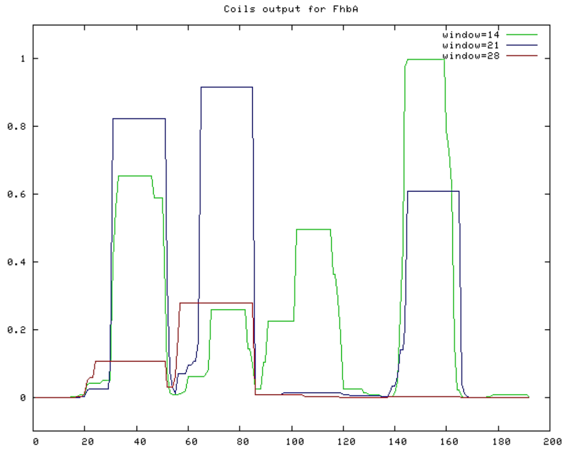

The predicted probability of α-helices and coiled coils structures within CbiA was assessed using ProtScale and COILS (www.expasys.org). COILS was performed with (2.5) and without weighting by using a 14-, 21-, and 28-amino-acid window.

Supplementary Figure S3

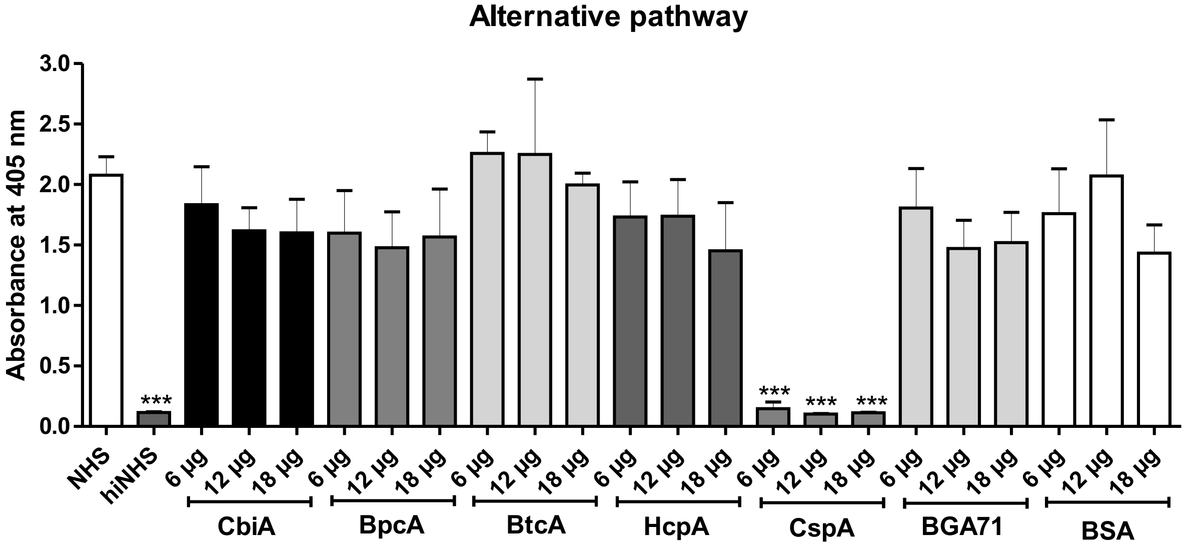

Complement inhibition of borrelial proteins on the alternative pathway. The inhibitory capacity of borrelial proteins on the alternative pathway was analyzed by Wielisa® (Euro Diagnostica, Malmö, Sweden). NHS diluted 1:18 was pre-incubated with increasing concentrations (6, 12, 18 μg/ml each) of borrelial proteins or BSA (negative control) and then added to microtiter plates coated with LPS. Following incubation, C5b-9 deposition was measured using an anti-C5b-9 mAb at 405 nm. Means from three independent experiments are shown and error bars correspond to SD. Raw data were analyzed using one-way ANOVA with post hoc Bonferroni correction. ***, *P* = .001, **, *P* = .01, *, *P* = .05.

Supplementary Figure S4

Uncropped gel and Western blots of figure 1

Panel 1B

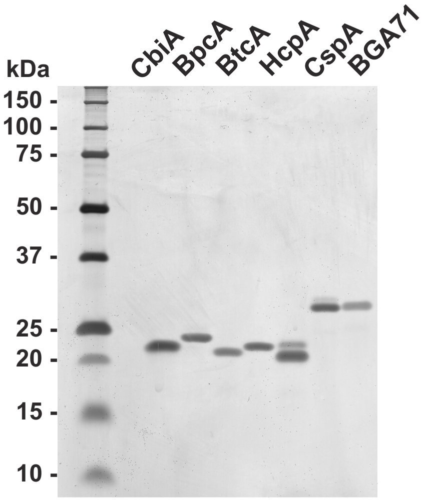

silver stained gel

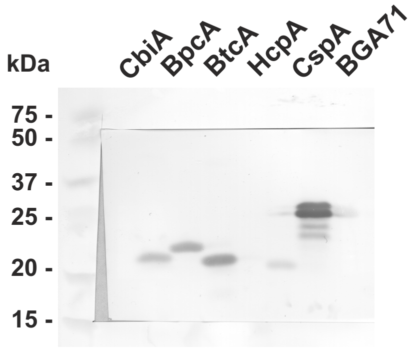

anti-FH

Panel 1D

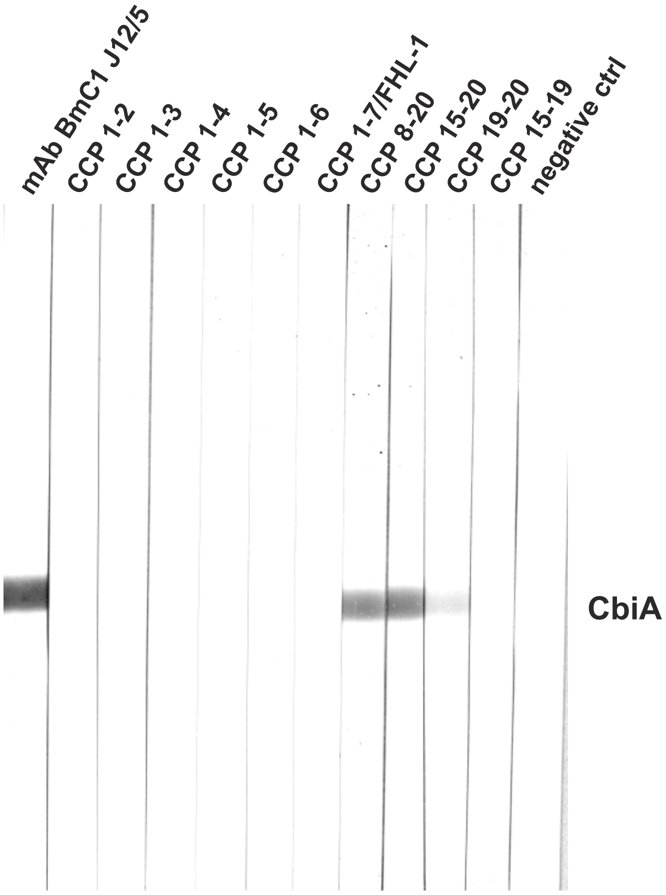

Panel 1E

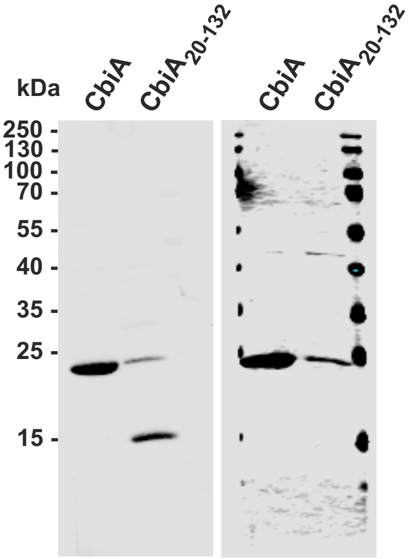

anti-His anti-FH

Supplementary Figure S5

Full-length Western blots of figure 2

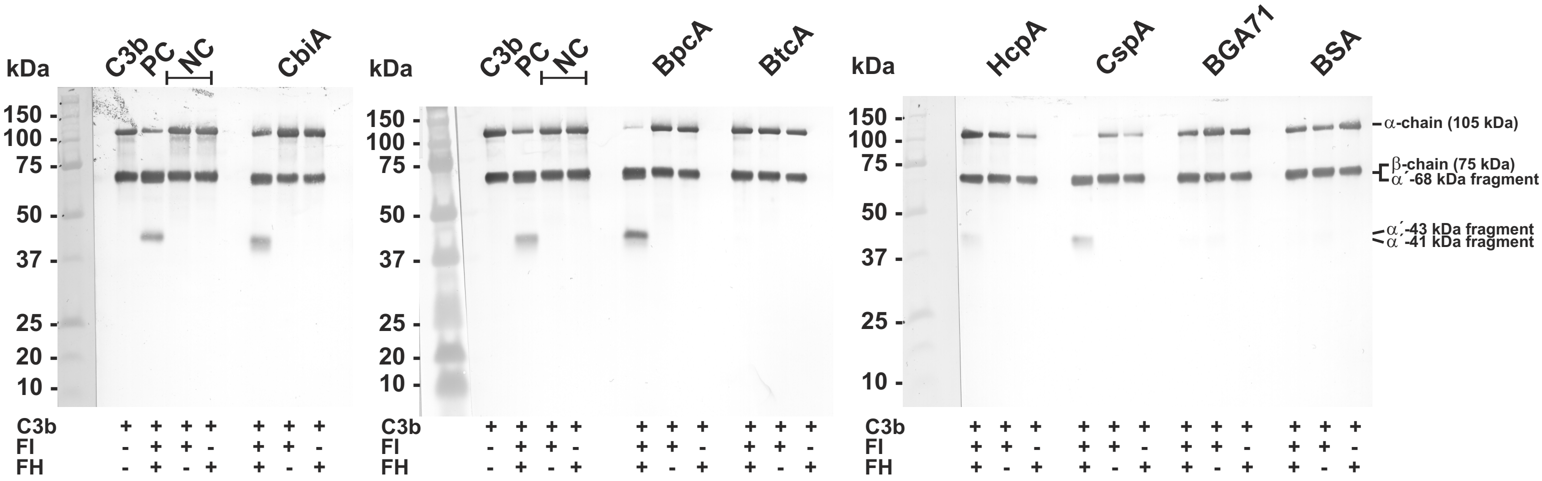

Supplementary Figure S6

Full-length Western blot of figure 5, panel B

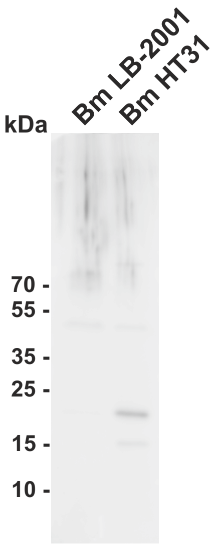

Supplementary Figure S7

Full-length Western blots of figure 6, panel D

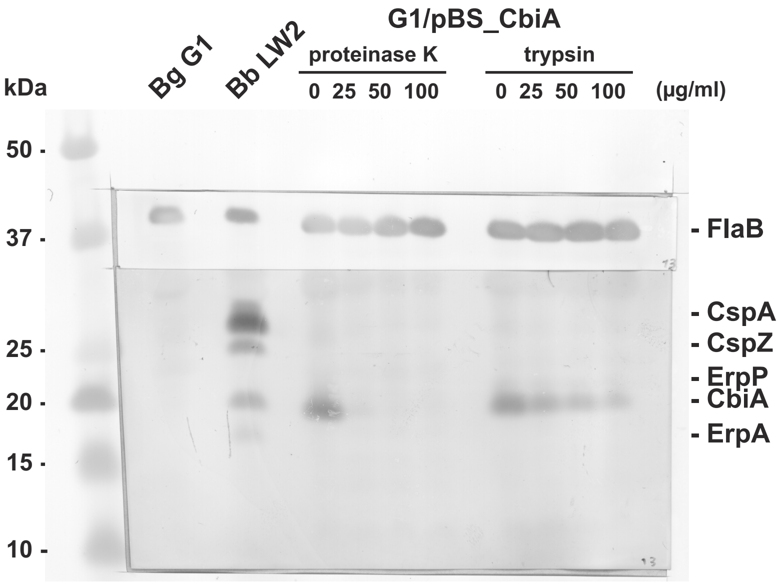

Supplementary Figure S8

Full-length Western blots of figure 7, panel B

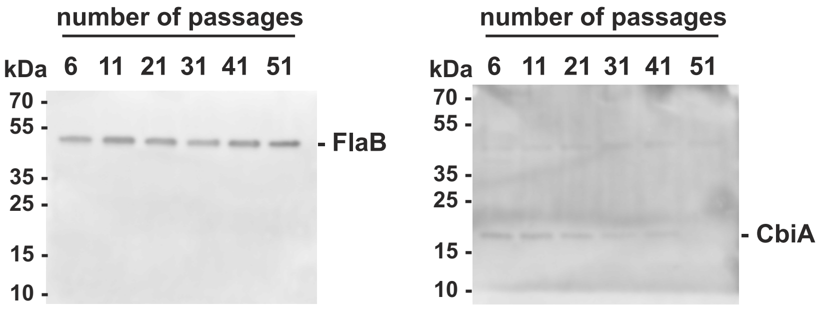

Supplementary Figure S9

Full-length Western blot of figure 8

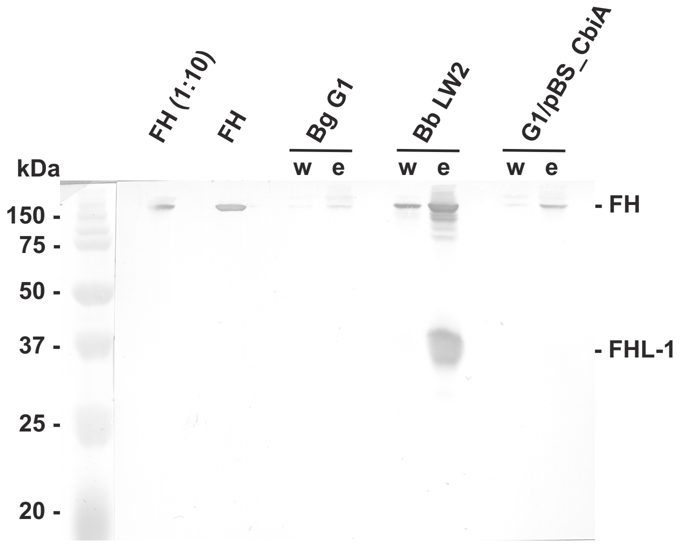

Supplementary Table S1. Oligonucleotides used in this study

Oligonucleotide	Sequence (5’-3’)[a]	Use in this work
cbiA F00	ATCTCAATATGCACTATATTTC	Cloning of *cbiA*
cbiA R0	CTGCAGTATCGATTTATATGTACTCTTC	Cloning of *cbiA*
cbiA Bam	CTATTATTAAACCTTATCGGATCCAATTTATTC	Cloning of *cbiA* in pQE-30 Xa; generation of His-tagged protein
cbiA Bam	CGAAGGATCCATGCACTATATTAAAAAATATC	Cloning of *cbiA* in pBSVA
cbiA Sph	CGTTTGCATGCACTCTTTCTCAGAAG	Cloning of *cbiA* in pBSVA
BtBam	GTCTTGTTGGATCCGGTTTATCAAG	Cloning of *btcA* in pQE-30 Xa;
BtSal	TATGTCGACTTAACTTTCTAAAAGTG	generation of His-tagged protein
glpQ FW	TCATGCTTTAAACAAGAAATG	qRT-PCR of *glpQ*
glpQ RV	TCTAGCTCGATTGGGAAATAATTG	qRT-PCR of *glpQ*
cbiA FW	AGAGCAAGCAAGCAAAAGTT	qRT-PCR of *cbiA*
cbiA RV	AAGCTTATAAGTGTGCCGTCATT	qRT-PCR of *cbiA*

[a], Sequences of specific restriction endonuclease recognition sites are underlined.
